# Adherence to Multitarget Stool DNA Testing in Individuals Aged 45–49 Years With Average Risk for Colorectal Cancer

**DOI:** 10.14309/ctg.0000000000000878

**Published:** 2025-06-26

**Authors:** Mallik Greene, Mark Camardo, A. Burak Ozbay, Michael Dore, A. Mark Fendrick, Paul Limburg

**Affiliations:** 1 Exact Sciences Corporation, Madison, Wisconsin, USA;; 2 Department of Medicine, Duke University, Durham, North Carolina, USA;; 3 Division of General Medicine, Department of Internal Medicine, University of Michigan, Ann Arbor, Michigan, USA.

**Keywords:** colorectal cancer, screening, mt-sDNA, adherence, young adults

## Abstract

**INTRODUCTION:**

Recommendations for colorectal cancer (CRC) screening have been updated to include individuals aged 45–49 years, addressing recent increases in CRC rates among young adults. The multitarget stool DNA (mt-sDNA) test is approved for average-risk individuals aged 45–49 years and is a noninvasive, at-home, stool-based option. This real-world study quantified mt-sDNA screening adherence rates for individuals aged 45–49 years.

**METHODS:**

This was a retrospective analysis of individuals aged 45–49 years with average risk for CRC and no history of mt-sDNA testing. Individuals received an mt-sDNA test kit based on a point-of-care order. Adherence was defined as the return of a kit resulting in a valid test result within 1 year of prescription.

**RESULTS:**

A total of 1,126,523 individuals were included. Most (58.6%) were female, and 62.2% received digital communications about the test through text message only. Overall, 68.9% returned their mt-sDNA kit within 1 year. Across provider types, adherence was highest for individuals who were prescribed the test by a gastroenterologist (75.5%); among payor types, adherence was highest for those enrolled in commercial insurance (70.9%). Adherence was lowest for individuals who did not receive any digital communications (54.9%) vs those who did. In a logistic regression analysis, digital communication and payor type were the strongest predictors of adherence.

**DISCUSSION:**

In this large real-world study, 68.9% of individuals aged 45–49 years returned an mt-sDNA CRC screening test within 1 year. Increasing digital communication may improve mt-sDNA screening adherence.

## INTRODUCTION

Colorectal cancer (CRC) is the second-leading cause of cancer-related death and the third most commonly diagnosed cancer in the United States among men and women combined (1,2). Alarmingly, over the last several decades, rates of CRC have increased in adults younger than 50 years, who now account for 10%–12% of all CRC cases (3). Specifically, the incidence of advanced-stage CRC has been increasing among individuals aged 45–49 years, highlighting a need for improved early detection for younger individuals (4). Most patients with early-onset CRC are considered average risk, and the underlying cause of the increase in CRC incidence remains unclear (5).

Screening programs that identify premalignant or early-stage neoplasia and allow for early intervention can improve patient outcomes, reduce mortality rates, and lower the financial burden of CRC (6–8). Several computational models demonstrate that screening of younger adults results in significant life-years gained (9,10).

Accordingly, in 2021, the US Preventive Services Task Force (USPSTF) updated its recommendations to include CRC screening for adults aged 45–49 years (6), shifting from earlier recommendations to begin screening at age 50 years (11). The American Cancer Society also recommends CRC screening for average-risk adults beginning at 45 years of age (12).

Adherence to screening recommendations can be difficult to achieve. A lack of provider recommendations regarding screening options for eligible individuals is a substantial barrier for those of all ages who might otherwise be unaware of their screening options (13). For adults younger than 50 years, factors such as finding childcare, getting time off work, or having a person available to drive them can limit access to colonoscopies (14). Adults in this age group may also believe they are not susceptible to CRC (15). In fact, although 68.8% of individuals aged 50 years and older report being up-to-date on CRC screening (16), only approximately 20% of eligible 45- to 49-year-olds report being up-to-date, with lower rates observed based on race, level of education, and medical insurance type (17). These factors may contribute to delayed diagnosis of CRC among young adults, resulting in higher rates of later-stage metastatic disease (5,15).

Several noninvasive, at-home, stool-based options are available for CRC screening, including the fecal immunochemical test (FIT) and the multitarget stool DNA (mt-sDNA) test, which provide alternatives for individuals who choose not to undergo a colonoscopy (12). However, adherence is a challenge even with stool-based tests. Analyses of adherence to FIT testing among individuals older than 50 years vary greatly by study, with findings ranging from 26% to 62% (18–20). Adherence rates are generally even lower for individuals aged 45–49 years (20,21).

Approval for the mt-sDNA test, which has a 95.2% specificity for detecting biomarkers of advanced colorectal neoplasia (22), was expanded in 2019 to include average-risk individuals aged 45–49 years (22). In a modeling analysis of mt-sDNA use, lowering the screening age to 45 years reduced the incidence of CRC and CRC mortality, resulted in 19 life-years gained per 1,000 patients, and reduced the overall financial burden of CRC (23).

Among individuals aged 50 years and older with an order for an mt-sDNA test, adherence has been reported as 66.8% for those with commercial insurance or Medicare (24) and 51.3% for those with Medicaid (25). However, for younger adults aged 45–49 years, only 20% were reported by the US National Health Interview Survey to be up-to-date with any CRC screening test in 2021, compared with 59% of individuals aged ≥45 years (26). Given recent recommendations for CRC screening in adults aged 45–49 years and factors that may inhibit adherence to CRC screening in this age group, there is a need for data on real-world mt-sDNA test adherence in individuals aged 45–49 years.

This real-world study examined mt-sDNA screening adherence rate of US adults aged 45–49 years at average risk for CRC. The impact of demographic variables, the provider's specialty, and digital outreach on adherence to mt-sDNA testing was also explored.

## METHODS

### Data sources

This was a retrospective analysis that drew on 2 data sources. The first source was a national multipayor claims database. The second source was aggregated laboratory data from Exact Sciences Laboratories LLC (Madison, WI), the exclusive national laboratory for mt-sDNA (Cologuard) testing. The ESL database includes information on all mt-sDNA laboratory data for nearly 6 million individuals per year, as well as data on laboratory test results, patient outreach, payor, and basic demographics.

The link between the national claims database and ESL data through tokenization created integrated data that provided complete information on mt-sDNA testing and results from ESL, as well as comprehensive health information derived from the claims database. Specifically, the race and ethnicity data were obtained from the claims database; the mt-sDNA adherence, time to adherence, positivity rates, and payor types were derived from the ESL database.

The data were deidentified and complied with the patient requirements of the US Health Insurance Portability and Accountability Act of 1996. The study was exempt from institutional review board approval because it used deidentified aggregate data.

### Study population and design

Data from January 1, 2016, to September 30, 2024, were used. The analysis included individuals in the United States aged 45–49 years with average risk for CRC and no history of mt-sDNA testing who received an mt-sDNA test kit from ESL based on a point-of-care order.

The index date was defined as the date of the first prescription order for the mt-sDNA test received within an index year. For each index year (2017–2023), individuals were required to be continuously enrolled in a health plan for a minimum of 2 years, including a 1-year baseline period for assessment of baseline characteristics and eligibility, and a 1-year follow-up period when individuals' adherence to CRC screening was assessed.

During the baseline period and study period, eligible individuals aged 45–49 years were required to be of average risk for CRC, defined as having no prior diagnosis indicating high risk, including diagnosis of CRC, inflammatory bowel disease or ulcerative colitis, familial polyposis syndromes, colonic adenomas, or colonic polyps. Individuals also had no documented family history of CRC or prior colectomy. All individuals for whom an mt-sDNA test was ordered were offered an enhanced navigation and support system that included automated and live phone calls, mailed letters, emails, and short message service (SMS) messages, which aimed to maximize screening adherence. Individuals could opt in to receive these digital communications through SMS only, email only, both SMS and email, or no digital communications (mail only). These preferences were communicated to ESL by their provider.

The analysis excluded individuals younger than 45 years or older than 49 years; those who were not covered by commercial, managed care organization, Medicare, Medicare Advantage, or Medicaid insurance plans; those who were considered high risk; those whose orders were not placed between January 1, 2017, and December 31, 2023; those who resided outside of the United States; and those who had missing information regarding their geographic region, urban/rural classification, or prescribing provider specialty.

### Outcome measures

Adherence to the mt-sDNA test was defined as the return of a kit that resulted in a valid test result (negative or positive) within 1 year from the index date. The time to test return was defined as the number of days between the date of kit shipment to the participant and the date of receipt by ESL of a valid test kit. Positivity was defined as a positive result on the mt-sDNA test.

### Statistical analysis

Descriptive statistics were used to summarize the baseline characteristics of the study population and the adherence rate. Adherence rates were compared within categories of characteristics including sex, race and ethnicity, preferred language, geographic location, provider specialty, type of health insurance (payor), and type of digital communication received. Frequencies, percentages, and *P* values from χ^2^ tests (and Fisher exact tests, where applicable) were reported.

Logistic regression analysis was used to assess demographics and characteristics associated with adherence to the mt-sDNA test (binary outcome: yes/no). Variables were included in the model if statistically significant differences were observed in the χ^2^ tests. Final regression results were obtained from a single multivariable logistic regression model adjusted for all covariates. Statistical analyses were conducted using SQL and SAS statistical software.

## RESULTS

**Figure 1.**
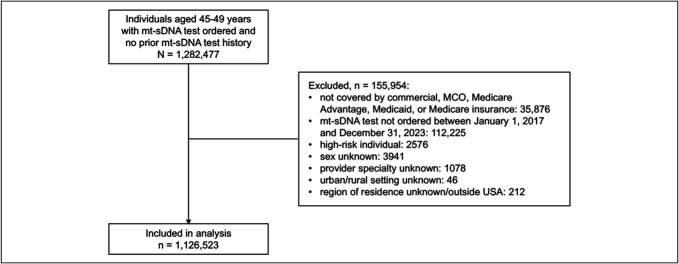
Disposition of individuals in the analysis. MCO, managed care organization; mt-sDNA, multitarget stool DNA.

A total of 1,282,477 individuals aged 45–49 years with no prior mt-sDNA testing history were identified in the data (Figure 1). Overall, 155,954 individuals were excluded, and the remaining 1,126,523 individuals were included in the analysis. Of these, 990,563 (87.9%) had commercial insurance (Table 1). Most individuals were female (659,670 [58.6%]), prescribed the test by a nurse practitioner (NP)/physician assistant (PA; 317,339 [28.2%]) or primary care physician (PCP; 673,494 [59.8%]), and received digital communications about the test via SMS only (700,677 [62.2%]; Table 1).

**Table 1.** Sociodemographic characteristics by payor type

n (%)	Overall (N = 1,126,523)	Commercial (N = 990,563)	MCO (N = 112,856)	Medicare Advantage (N = 16,137)	Medicaid (N = 4,355)	Medicare (N = 2,612)
Sex						
Female	659,670 (58.6)	573,918 (57.9)	72,394 (64.2)	9,206 (57.1)	2,759 (63.4)	1,393 (53.3)
Male	466,853 (41.4)	416,645 (42.1)	40,462 (35.9)	6,931 (43.0)	1,596 (36.7)	1,219 (46.7)
Race and ethnicity						
Asian or Pacific Islander	50,738 (4.5)	44,544 (4.5)	5,481 (4.9)	430 (2.7)	212 (4.9)	71 (2.7)
Black or African American	96,606 (8.6)	69,980 (7.1)	22,301 (19.8)	3,193 (19.8)	697 (16.0)	435 (16.7)
Hispanic or Latino	107,631 (9.6)	89,214 (9.0)	15,628 (13.9)	2,020 (12.5)	507 (11.6)	262 (10.0)
White	431,443 (38.3)	366,525 (37.0)	52,110 (46.2)	8,820 (54.7)	2,226 (51.1)	1,762 (67.5)
Unknown	440,105 (39.1)	420,300 (42.4)	17,336 (15.4)	1,674 (10.4)	713 (16.4)	82 (3.1)
Preferred language						
English	273,382 (24.3)	232,579 (23.5)	33,714 (29.9)	5,277 (32.7)	1,065 (24.5)	747 (28.6)
Spanish	16,706 (1.5)	12,558 (1.3)	3,805 (3.4)	224 (1.4)	102 (2.3)	17 (0.7)
Other	4,165 (0.4)	2,495 (0.3)	1,536 (1.4)	39 (0.2)	80 (1.8)	15 (0.6)
Unknown	832,270 (73.9)	742,931 (75.0)	73,801 (65.4)	10,597 (65.7)	3,108 (71.4)	1,833 (70.2)
Region						
Northeast	227,663 (20.2)	188,585 (19.0)	32,437 (28.7)	4,002 (24.8)	1,492 (34.3)	1,147 (43.9)
Midwest	302,594 (26.9)	262,192 (26.5)	34,932 (31.0)	3,990 (24.7)	845 (19.4)	635 (24.3)
South	463,907 (41.2)	427,199 (43.1)	29,654 (26.3)	5,719 (35.4)	798 (18.3)	537 (20.6)
West	132,359 (11.8)	112,587 (11.4)	15,833 (14.0)	2,426 (15.0)	1,220 (28.0)	293 (11.2)
Urban/rural classification						
Metropolitan	968,743 (86.0)	858,548 (86.7)	91,130 (80.8)	13,627 (84.5)	3,18 (76.19)	2,120 (81.16)
Micropolitan	93,053 (8.3)	78,032 (7.9)	12,736 (11.3)	1,514 (9.4)	469 (10.8)	302 (11.6)
Rural	24,674 (2.2)	20,493 (2.1)	3,483 (3.1)	342 (2.1)	281 (6.5)	75 (2.9)
Small town	40,053 (3.6)	33,490 (3.4)	5,507 (4.9)	654 (4.1)	287 (6.6)	115 (4.4)
Provider specialty						
GI	9,804 (0.9)	7,802 (0.8)	1,538 (1.4)	286 (1.8)	72 (1.7)	106 (4.1)
NP/PA	317,339 (28.2)	270,519 (27.3)	39,430 (34.9)	4,967 (30.8)	1,748 (40.1)	675 (25.8)
OB/GYN	70,699 (6.3)	66,631 (6.7)	3,484 (3.1)	386 (2.4)	144 (3.3)	54 (2.1)
PCP	673,494 (59.8)	600,020 (60.6)	60,329 (53.5)	9,444 (58.5)	2,065 (47.4)	1,636 (62.6)
Other	55,187 (4.9)	45,591 (4.6)	8,075 (7.2)	1,054 (6.5)	326 (7.5)	141 (5.4)
Digital communication						
SMS + email	283,114 (25.1)	252,667 (25.5)	24,477 (21.7)	4,451 (27.6)	940 (21.6)	579 (22.2)
Email only	24,734 (2.2)	20,352 (2.1)	3,399 (3.0)	748 (4.6)	145 (3.3)	90 (3.5)
SMS only	700,677 (62.2)	617,286 (62.3)	70,688 (62.6)	8,666 (53.7)	2,654 (60.9)	1,383 (53.0)
None	117,998 (10.5)	100,258 (10.1)	14,292 (12.7)	2,272 (14.1)	616 (14.1)	560 (21.4)

GI, gastroenterologist; GYN, gynecologist; MCO, managed care organization; NP, nurse practitioner; OB, obstetrician; PA, physician assistant; PCP, primary care physician; SMS, short message service.

**Figure 2.**
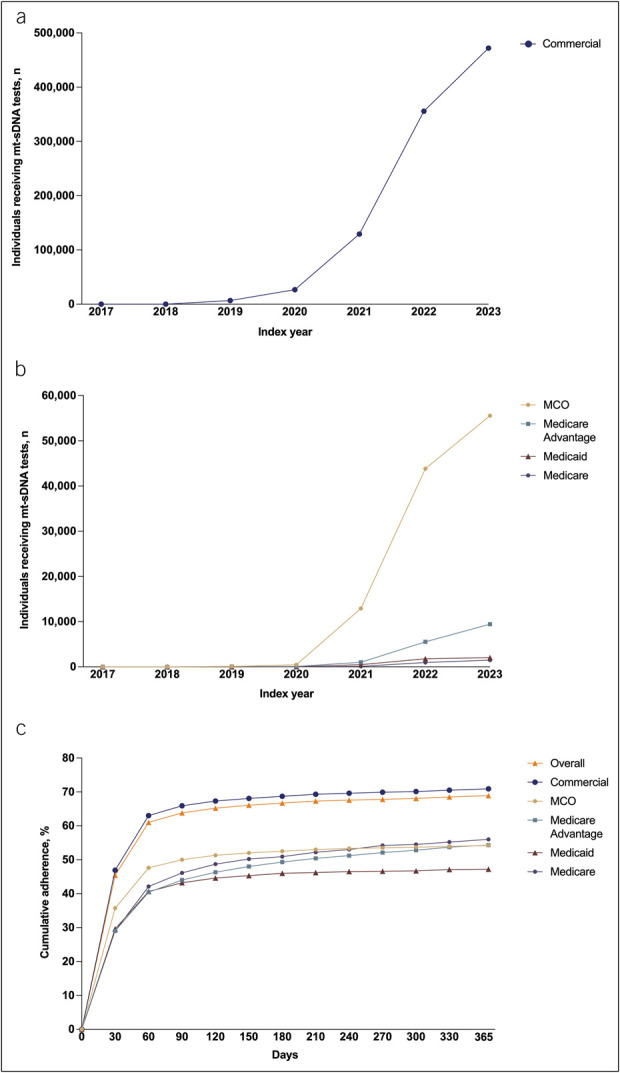
Number of mt-sDNA tests received by year and participants, and mt-sDNA cumulative adherence. (**a**) Number of mt-sDNA tests received by individuals with commercial insurance. (**b**) Number of mt-sDNA tests received by those with other insurance types. (**c**) Cumulative adherence over time by payor type. MCO, managed care organization; mt-sDNA, multitarget stool DNA.

Overall, 775,714 (68.9%) individuals returned their mt-sDNA kit within 1 year (Table 2). The number of mt-sDNA tests received increased each year after commercial availability of the mt-sDNA test and after CRC screening was recommended for individuals aged 45–49 years in 2021 (Figure 2a and b). Among the variables assessed in the overall population, adherence rates were highest among individuals whose test was prescribed by a gastroenterologist (GI; 75.5%) and lowest among individuals who did not receive digital communications (54.9%). Adherence rates were higher among individuals enrolled in commercial insurance (70.9%) than other insurance types (ranging from 47.2% for Medicaid enrollees to 56.0% for Medicare; Table 2); however, the rate at which mt-sDNA tests were returned was numerically comparable across payor types, with most tests being returned within 60 days (Figure 2c).

**Table 2.** Adherence rates by payor type

n (%); *P* values by category^a^	Overall (N = 1,126,523)	Commercial (N = 990,563)	MCO (N = 112,856)	Medicare Advantage (N = 16,137)	Medicaid (N = 4,355)	Medicare (N = 2,612)
Overall	775,714 (68.9)	702,263 (70.9)	61,163 (54.2)	8,770 (54.3)	2,055 (47.2)	1,463 (56.0)
Sex	<0.0001	<0.0001	0.000633	<0.0001	0.11117	0.67947
Female	447,637 (67.9)	401,753 (70.0)	38,961 (53.8)	4,872 (52.9)	1,276 (46.2)	775 (55.6)
Male	328,077 (70.3)	300,510 (72.1)	22,202 (54.9)	3,898 (56.2)	779 (48.7)	688 (56.4)
Race and ethnicity	<0.0001	<0.0001	<0.0001	<0.0001	<0.0001	0.005736
Asian or Pacific Islander	35,582 (70.1)	31,648 (71.1)	3,489 (63.6)	267 (62.2)	128 (59.8)	50 (70.4)
Black or African American	60,226 (62.3)	46,477 (66.4)	11,626 (52.1)	1,625 (50.8)	275 (39.5)	223 (51.1)
Hispanic or Latino	68,384 (63.5)	59,073 (66.2)	7,976 (51.1)	1,006 (49.8)	195 (38.5)	134 (51.0)
White	299,745 (69.5)	263,988 (72.0)	28,718 (55.1)	4,949 (56.1)	1,083 (48.7)	1,007 (57.2)
Unknown	311,777 (70.8)	301,077 (71.6)	9,354 (54.0)	923 (55.2)	374 (52.2)	49 (60.5)
Preferred language	<0.0001	<0.0001	<0.0001	<0.0001	0.046562	0.69581
English	177,434 (64.9)	158,424 (68.1)	15,502 (46.0)	2,636 (50.0)	468 (43.9)	404 (54.2)
Spanish	10,882 (65.1)	8,469 (67.4)	2,222 (58.5)	128 (57.4)	53 (52.0)	10 (58.8)
Other	2,903 (69.8)	1,793 (71.9)	1,033 (67.3)	25 (64.1)	43 (53.8)	9 (60.0)
Unknown	584,495 (70.2)	533,577 (71.8)	42,406 (57.5)	5,981 (56.4)	1,491 (48.0)	1,040 (56.7)
Region	<0.0001	<0.0001	<0.0001	0.002369	<0.0001	0.000847
Northeast	149,819 (65.8)	129,252 (68.5)	16,815 (51.8)	2,256 (56.4)	860 (57.6)	636 (55.5)
Midwest	214,708 (71.0)	192,135 (73.3)	19,890 (56.9)	2,100 (52.6)	236 (27.9)	347 (54.6)
South	316,757 (68.3)	297,486 (69.6)	15,628 (52.7)	3,064 (53.6)	294 (37.0)	285 (52.8)
West	94,430 (71.3)	83,390 (74.1)	8,830 (55.7)	1,350 (55.7)	665 (54.4)	195 (66.8)
Urban/rural classification	0.073253	<0.0001	0.046125	0.001747	0.001833	0.02087
Metropolitan	666,864 (68.8)	607,452 (70.8)	49,337 (54.1)	7,331 (53.8)	1,529 (46.1)	1,215 (57.3)
Micropolitan	63,999 (68.8)	55,834 (71.6)	6,916 (54.3)	884 (58.4)	218 (46.5)	147 (48.7)
Rural	17,146 (69.5)	14,781 (72.1)	1,971 (56.6)	206 (60.2)	143 (50.9)	45 (60.0)
Small town	27,705 (69.2)	24,164 (72.2)	2,962 (53.8)	357 (54.6)	164 (57.1)	58 (50.4)
Provider specialty	<0.0001	<0.0001	<0.0001	<0.0001	0.005525	0.086248
GI	7,400 (75.5)	6,064 (77.7)	1,025 (66.7)	198 (69.2)	40 (55.6)	73 (68.9)
NP/PA	205,871 (64.9)	180,721 (66.8)	21,420 (54.3)	2,532 (51.0)	824 (47.2)	374 (55.5)
OB/GYN	50,505 (71.4)	48,038 (72.1)	2,157 (61.9)	209 (54.1)	74 (51.0)	27 (50.0)
PCP	475,892 (70.7)	436,067 (72.7)	32,665 (54.2)	5,313 (56.3)	935 (45.3)	912 (55.7)
Other	36,046 (65.3)	31,373 (68.8)	3,896 (48.2)	518 (49.2)	182 (55.3)	77 (54.6)
Digital communication	<0.0001	<0.0001	<0.0001	<0.0001	0.00134	0.83253
SMS + email	202,255 (71.4)	185,060 (73.2)	13,859 (56.6)	2,536 (57.0)	469 (49.7)	331 (57.1)
Email only	15,820 (64.0)	13,611 (66.9)	1,723 (50.7)	377 (50.4)	62 (42.5)	47 (52.2)
SMS only	492,835 (70.3)	447,248 (72.5)	38,811 (54.9)	4,732 (54.6)	1,275 (48.1)	769 (55.7)
None	64,804 (54.9)	56,344 (56.2)	6,770 (47.4)	1,125 (49.5)	249 (40.5)	316 (56.3)

GI, gastroenterologist; GYN, gynecologist; MCO, managed care organization; NP, nurse practitioner; OB, obstetrician; PA, physician assistant; PCP, primary care physician; SMS, short message service.

a *P* values are based on χ^2^ tests by category.

Overall, the mean (SD) time to return the test was 30.2 (46.33) days (Table 3). Numerically (with no statistical comparisons performed), the time to return the test was longer for individuals who received no or email-only digital communications vs those who received SMS messages. Among practitioner specialties, the test was returned fastest when ordered by a GI and slowest when ordered by an obstetrician/gynecologist (Table 3).

**Table 3.** Time to adherence among individuals with returned tests by payor type (days)

Mean (SD)	Overall (N = 775,714)	Commercial (N = 702,263)	MCO (N = 61,163)	Medicare Advantage (N = 8,770)	Medicaid (N = 2,055)	Medicare (N = 1,463)
Overall	30.2 (46.33)	30.3 (46.56)	29.4 (44.80)	28.2 (40.35)	28.6 (39.93)	29.0 (40.12)
Sex						
Female	31.3 (47.57)	31.4 (47.85)	30.1 (45.54)	29.1 (41.29)	29.3 (41.97)	31.0 (45.19)
Male	28.7 (44.54)	28.8 (44.73)	28.2 (43.43)	27.2 (39.10)	27.3 (36.27)	26.6 (33.24)
Race and ethnicity						
Asian or Pacific Islander	28.5 (44.59)	29.1 (45.69)	23.9 (34.02)	24.8 (28.28)	20.8 (18.71)	41.0 (69.73)
Black or African American	28.4 (44.38)	28.2 (44.38)	29.0 (44.93)	27.4 (38.60)	28.9 (42.98)	34.0 (55.19)
Hispanic or Latino	29.4 (43.89)	29.6 (44.47)	28.5 (40.57)	26.9 (35.93)	22.5 (22.99)	28.3 (48.41)
White	30.9 (47.30)	31.0 (47.55)	30.3 (46.32)	29.2 (42.31)	31.5 (44.39)	27.4 (32.61)
Unknown	30.2 (46.45)	30.2 (46.48)	30.0 (46.64)	27.2 (40.00)	25.6 (35.36)	27.8 (32.09)
Preferred language						
English	29.4 (44.38)	29.4 (44.45)	29.6 (44.23)	27.4 (39.16)	32.1 (47.40)	35.8 (47.55)
Spanish	27.3 (36.99)	27.6 (37.97)	26.5 (33.81)	25.1 (29.19)	17.4 (12.79)	19.9 (25.49)
Other	23.9 (35.49)	22.6 (32.61)	26.7 (40.82)	13.2 (6.71)	22.3 (21.49)	11.0 (6.38)
Unknown	30.5 (47.10)	30.6 (47.32)	29.6 (45.57)	28.8 (41.12)	28.1 (38.35)	26.5 (36.76)
Region						
Northeast	31.7 (50.53)	32.1 (51.14)	29.6 (47.34)	28.7 (43.15)	27.5 (38.90)	30.5 (40.14)
Midwest	30.2 (47.31)	30.4 (47.63)	28.8 (44.70)	27.4 (41.14)	31.5 (45.65)	27.0 (43.54)
South	28.7 (43.85)	28.8 (44.02)	27.8 (42.10)	26.7 (37.41)	23.5 (33.95)	27.2 (33.89)
West	32.6 (45.03)	32.5 (45.20)	33.2 (44.44)	32.4 (40.45)	31.1 (41.29)	29.9 (42.05)
Urban/rural classification						
Metropolitan	30.3 (46.76)	30.4 (47.01)	29.4 (44.95)	28.3 (40.03)	27.3 (37.92)	28.5 (39.65)
Micropolitan	29.5 (43.82)	29.6 (44.01)	29.4 (43.67)	24.5 (29.93)	33.9 (44.73)	26.3 (41.73)
Rural	31.2 (44.96)	31.2 (44.87)	30.8 (43.67)	36.1 (57.92)	32.7 (51.86)	39.7 (43.18)
Small town	29.0 (42.27)	28.9 (41.68)	29.1 (45.64)	32.1 (53.88)	29.3 (39.09)	36.3 (42.12)
Provider specialty						
GI	26.0 (37.54)	26.6 (38.76)	22.9 (31.57)	26.0 (33.51)	20.6 (14.58)	27.3 (26.12)
NP/PA	29.6 (44.42)	29.7 (44.62)	28.9 (43.35)	28.7 (42.09)	26.2 (33.71)	25.5 (40.16)
OB/GYN	31.7 (48.65)	31.6 (48.57)	32.7 (51.43)	26.7 (45.89)	23.9 (21.98)	33.5 (41.96)
PCP	30.4 (47.07)	30.4 (47.30)	29.6 (45.34)	28.0 (39.01)	29.9 (42.16)	30.8 (42.01)
Other	30.2 (45.46)	30.1 (45.25)	30.7 (46.84)	29.7 (45.07)	35.8 (58.25)	22.6 (18.55)
Digital communication						
SMS + email	28.6 (43.87)	28.6 (43.93)	28.8 (44.34)	25.9 (35.75)	31.1 (47.89)	28.3 (41.10)
Email only	33.6 (51.43)	34.1 (52.18)	29.9 (46.54)	31.4 (48.31)	28.4 (31.75)	28.3 (37.47)
SMS only	29.8 (45.84)	29.9 (46.04)	29.5 (44.55)	28.0 (39.61)	27.5 (37.37)	26.1 (34.79)
None	37.3 (54.89)	38.2 (55.90)	30.2 (46.68)	33.6 (48.89)	29.2 (38.16)	36.8 (49.74)

GI, gastroenterologist; GYN, gynecologist; MCO, managed care organization; NP, nurse practitioner; OB, obstetrician; PA, physician assistant; PCP, primary care physician; SD, standard deviation; SMS, short message service.

Among the 775,714 individuals who returned their mt-sDNA kit within 1 year, 48,033 (6.2%) had a positive result (Table 4). There was a significant effect of sex and race and ethnicity on the rate of positive screening results: male individuals had a higher rate of positive results (7.1%) than female individuals (5.5%) and Asian or Pacific Islander individuals had the lowest rate of positive results (3.2%), while White individuals had the highest rate of positive results (7.2%). It is important to note that among those who returned their tests, individuals of unknown race and ethnicity made up the second-largest racial group (18,914 individuals).

**Table 4.** Positivity rates on returned mt-sDNA tests

Characteristic	Individuals with returned tests (N = 775,714), n (%)
Overall	48,033 (6.2)
Sex	*P* < 0.0001^a^
Female	24,646 (5.5)
Male	23,387 (7.1)
Race and ethnicity	*P* < 0.0001^a^
Asian or Pacific Islander	1,143 (3.2)
Black or African American	3,233 (5.4)
Hispanic or Latino	3,141 (4.6)
White	21,602 (7.2)
Unknown	18,914 (6.1)

mt-sDNA, multitarget stool DNA.

a *P* values are based on χ^2^ tests by category.

**Figure 3.**
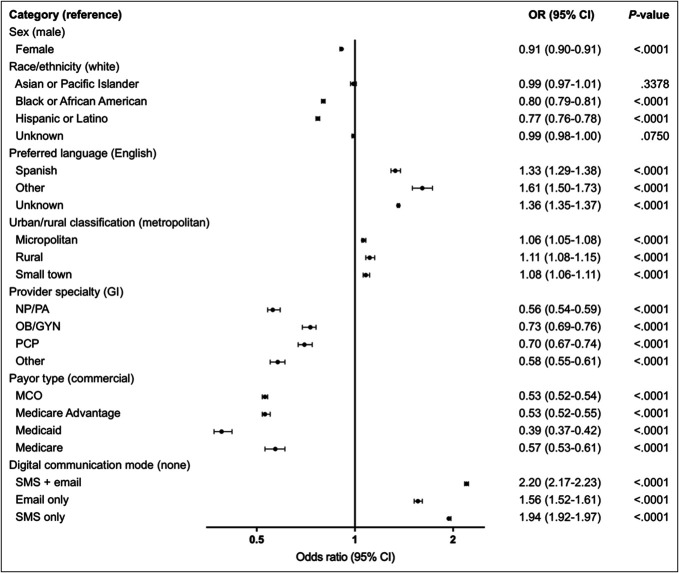
Logistic regression for factors associated with adherence. The x-axis is depicted on a log base 2 scale. CI, confidence interval; GI, gastroenterologist; GYN, gynecologist; MCO, managed care organization; NP, nurse practitioner; OB, obstetrician; OR, odds ratio; PA, physician assistant; PCP, primary care physician; SMS, short message service.

In the logistic regression analysis, digital communication mode and payor type were the strongest predictors of adherence (Figure 3). After adjusting for payor type, sex, race and ethnicity, preferred language, urban/rural classification, and provider type, individuals who received both SMS and email communications were 2.2 times more likely to return their mt-sDNA test than were individuals who received no digital communications. In addition, after adjusting for the other predictors, individuals on Medicaid were 61% less likely to adhere and individuals enrolled in other insurance types were between 43% and 47% less likely to adhere than individuals enrolled in commercial insurance (Figure 3). Regarding the specialty of the prescribing clinician, individuals whose test was ordered by an NP/PA had the lowest odds of returning the test (44% lower than individuals who saw a GI). Black or African American and Hispanic or Latino individuals were less likely to return their mt-sDNA tests than were White individuals; however, Spanish-speaking individuals had higher odds of adhering than English-speaking individuals (Figure 3).

## DISCUSSION

In this analysis of more than 1 million individuals aged 45–49 years at average risk for CRC who were prescribed an mt-sDNA test, the overall adherence rate was 68.9%. This is consistent with earlier analyses of mt-sDNA testing, which found 66.8% adherence for adults older than 50 years (24) and 71.3% for individuals aged 45–85 years (27). The rate of adherence in this study is also generally higher than reported levels of FIT adherence for individuals younger than 50 years. In an outreach program where FITs were mailed to eligible participants aged 45–49 years, only 18% returned their FIT within 6 months (21). Among Black participants included in a separate CRC screening outreach program, 40.5% of those aged 45–49 years completed a FIT, compared with 49.5% of participants aged 50–54 years (20). In another analysis among individuals aged 50 years or older with a positive mt-sDNA or FIT result, 78.8% and 54.5%, respectively, completed a follow-up colonoscopy, and the time to follow-up colonoscopy was faster for the mt-sDNA group (28). In the context of these earlier findings, adherence to the mt-sDNA test was robust in this large analysis of individuals aged 45–49 years.

Improved uptake of at-home, stool-based screening tests has important implications in a healthcare system that is challenged by delivering the needed CRC screening services, notably in rural settings (29,30). The COVID-19 pandemic disrupted access to CRC screening and led to a backlog in colonoscopy services (31). Recent recommendations that 45- to 49-year-olds be screened for CRC have further expanded the pool of individuals requiring screening services (30). Approaches have been suggested to optimize the use of at-home CRC screening tests alongside colonoscopy (27,32,33). In recent years, mt-sDNA screening rates have increased significantly, and rates of FIT have decreased; among 45- to 49-year-olds, utilization of both colonoscopy and mt-sDNA have increased, and rates of FIT use have decreased (34). The rates of screening for younger individuals likely increased because of the updated USPSTF guidelines (6), and the strong adherence of individuals aged 45–49 years to mt-sDNA testing in this study, supported by robust patient navigation, suggests that the mt-sDNA test is an effective option for continuing to improve the rates of CRC screening.

Although overall adherence to the mt-sDNA screening test was strong in this study, certain subpopulations had lower adherence. African American and Latino individuals in this study had adherence rates of 62.3% and 63.5%, respectively, and had lower odds of adherence compared with White individuals. It has been found that African American patients have an overall lower rate of CRC screening and are less likely to receive a timely follow-up to a positive test, leading to higher rates of later-stage metastatic disease on diagnosis (26). For Latino individuals, a large proportion of whom are immigrants, factors such as not having a PCP, not speaking English, and the overall complexity of the medical system in the United States can contribute to lower adherence to screening (35). Importantly, when Latino individuals were provided with a Spanish-language screening initiative, CRC screening adherence increased by 4.7% (36). For Black and Hispanic individuals, reducing the barriers to effective CRC screening adherence is an important step to improving CRC outcomes.

In this study, individuals who received both SMS and email communication from the manufacturer were 71.4% adherent, whereas those who received only mail communications were 54.9% adherent. In regression analysis, individuals who received both email and text reminders from the test provider had more than two-fold greater odds of adherence than individuals receiving no digital communications. Although this study cannot determine the underlying cause of this improved adherence, it may be a result of more frequent reminders, greater accessibility to instructional materials, or that those who opted in to digital communications are more inherently motivated. An earlier analysis that examined the effect of digital communication on mt-sDNA test adherence had similar findings, with the highest adherence (72.9%) among those who received outreach through SMS and email and the lowest adherence (63.4%) among those who received no digital communications (37). Thus, accumulating evidence demonstrates that the digital communications offered with the mt-sDNA test support greater adherence to CRC screening.

Adherence in this study was also affected by the provider that ordered the mt-sDNA test: adherence was 75.5% when the test was ordered by a GI, compared with ∼71% when ordered by an obstetrician/gynecologist or PCP, and 64.9% when ordered by an NP/PA. Prescription by a GI was also identified as a strong predictor of adherence in regression analysis, and tests were also returned more quickly, on average, when ordered by a GI. Prior analyses have similarly reported a higher rate of adherence for mt-sDNA tests prescribed by a GI (24,29). Although all individuals in this study were at average risk for CRC, individuals who were referred to a GI may have had heightened concern for their health or have been more motivated to complete their screening. GIs may also have provided more targeted or comprehensive education about the benefits of CRC screening; CRC screening education is associated with better adherence (38).

In this study, individuals' adherence to mt-sDNA testing also varied greatly by payor type (commercial insurance, 70.9%; managed care organization, 54.2%; Medicare Advantage, 54.3%; Medicaid 47.2%; Medicare 56.0%). Having commercial insurance was a strong predictor of screening adherence compared with noncommercial insurance. The number of individuals with Medicaid or Medicare coverage in this study was relatively small (4,355 and 2,612, respectively), raising the possibility that these individuals were not representative of all adults enrolled in those insurance plans. Specifically, patients aged 45–49 years who receive Medicare insurance are either disabled or have serious chronic illness, conditions that could affect their adherence to CRC screening. The finding that individuals enrolled in Medicaid were less likely to adhere to CRC screening is consistent with earlier research (25,29,39) and may also have influenced our findings regarding provider type; for example, individuals on Medicaid may have been more likely to be cared for by an NP or PA (40). Future research investigating the impact of payor type on CRC screening adherence may highlight the specific barriers for publicly insured individuals aged 45–49 and can inform strategies to improve screening adherence among younger individuals without commercial insurance.

Increasing CRC screening adherence is an important public health goal. The addition of patient incentives (i.e., something of monetary value) to the screening process may increase screening participation (41), but findings are mixed (42). Screening reminders, updates about testing status, and other navigation services that guide individuals through the screening process improve screening participation (42). These services are automatically included as part of the mt-sDNA testing process. In addition, providers who give targeted education regarding the mt-sDNA test—including instructions for completing the test and information about the cost and potentially necessary follow-ups—have a higher rate of screening compliance (38).

A limitation of this analysis is the lack of a control group. Because the changes in screening recommendations for younger adults are relatively recent, the data regarding screening adherence are somewhat limited. Direct comparisons of mt-sDNA with other screening modalities for adults aged 45–49 years would be helpful for determining the most productive approach to improving CRC screening adherence in this age group. Additional limitations include the retrospective nature of the study and the self-selection of several patient variables. For instance, an individual's choice to see a particular provider (e.g., a GI) or to receive digital communications may reflect a personal willingness to complete CRC screening, associations that are not reflected in this analysis. Similarly, other characteristics—such as regional geography, socioeconomic status, or level of education—could affect personal motivation to complete screening. It is also unclear how many individuals aged 45–49 from the 2 source databases were eligible for screening and either offered an alternative screening method or not offered screening at all. The reasons for this may relate to patient or provider characteristics that could influence adherence. Future research that investigates the reasons for individual or provider choices regarding screening can better identify the factors that motivate younger adults to complete CRC screening. In addition, this analysis did not include individuals without medical insurance coverage, and there was a substantial amount of missing demographic data regarding race and ethnicity and preferred language, all of which may influence an individual's ability to complete CRC screening.

In conclusion, screening for CRC is crucial to reduce the health and financial burdens of CRC. For younger adults, who are experiencing increasing rates of CRC, in particular, the mt-sDNA test is a convenient, home-based option for screening. Screening adherence in this study was 68.9%, which compared favorably with previous research in older cohorts. Regression analysis revealed lower adherence for Black and Latino individuals and higher adherence for individuals who received their test from a GI or had commercial health insurance. Digital communication also improved adherence. Strong adherence to the mt-sDNA screening test among 45- to 49-year-old adults in this study suggests that it is an effective approach to improve CRC screening rates in a healthcare system that finds providing the needed screening services challenging.

## CONFLICTS OF INTEREST

**Guarantor of the article**: Mallik Greene, PhD, DBA, BPharm.

**Specific author contributions:** M.G.: approves the manuscript; contributed in the categories of conceptualization, formal analysis, funding acquisition, investigation, methodology, project administration, resources, software, supervision, validation, visualization, and writing (review & editing). M.C.: approves the manuscript; contributed in the categories of data curation, formal analysis, investigation, methodology, validation, visualization, and writing (review & editing). A.B.O.: approves the manuscript; contributed in the categories of conceptualization, funding acquisition, investigation, methodology, resources, supervision, and writing (review & editing). M.D.: approves the manuscript; contributed in the categories of conceptualization, investigation, methodology, supervision, validation, visualization, and writing (review & editing). A.M.F.: approves the manuscript; contributed in the categories of conceptualization, investigation, methodology, supervision, validation, visualization, and writing (review & editing). P.L.: approves the manuscript; contributed in the categories of conceptualization, funding acquisition, investigation, methodology, resources, supervision, and writing (review & editing).

**Financial support:** This study was funded by Exact Sciences Corporation. They were involved in the study design, data collection and analysis, and interpretation of the data—as well as in the writing of the report.

**Potential competing interests:** M.G., M.C., A.B.O., and P.L. are employees of Exact Sciences Corporation and own stock/stock options. A.M.F. has been a consultant for AbbVie, Amgen, Centivo, Community Oncology Association, Covered California, EmblemHealth, Exact Sciences, Freedman HealthCare, GRAIL, Harvard University, Health & Wellness Innovations, Health at Scale Technologies, MedZed, Penguin Pay, Risalto Health, Sempre Health, the State of Minnesota, the US Department of Defense, Virginia Center for Health Innovation, Wellth, and Zansors; has received research support from the Agency for Healthcare Research and Quality, the Gary and Mary West Health Policy Center, Arnold Ventures, the National Pharmaceutical Council, the Patient-Centered Outcomes Research Institute, Pharmaceutical Research and Manufacturers of America, the Robert Wood Johnson Foundation, the State of Michigan, and the Centers for Medicare and Medicaid Services. M.D. is an Associate Professor of Medicine at Duke University.

**IRB statement:** The study was considered exempt research under 45 CFR § 46.104(d)(4) as it involved only the secondary use of data that were deidentified (aggregate) in compliance with the US Health Insurance Portability and Accountability Act of 1996, specifically, 45 CFR § 164.514.

**Data availability statement:** The data that support the findings of this study are available from Exact Sciences Laboratories LLC. Restrictions apply to the availability of these data, which were used under license for this study. Data are available from the authors with the permission of Exact Sciences Laboratories LLC.

**Study Highlights**

**WHAT IS KNOWN**

✓ Colorectal cancer incidence has increased in adults younger than 50 years.
✓ Colorectal cancer screening adherence remains low in adults aged 45–49 years.

**WHAT IS NEW HERE**

✓ Screening adherence was nearly 70% in persons aged 45–49 years.
✓ Higher adherence was seen in individuals who received their test from a gastroenterologist.
✓ Digital communication and payor type were the strongest predictors of adherence.

## ABBREVIATIONS:

CRC: colorectal cancer
ESL: Exact Sciences Laboratories
FIT: fecal immunochemical test
GI: gastroenterologist
mt-sDNA: multitarget stool DNA
NP: nurse practitioner
PA: physician assistant
SMS: short message service
USPSTF: US Preventative Services Task Force
